# Microwave-Assisted Heating Reactions of *N*-Acetylglucosamine (GlcNAc) in Sulfolane as a Method Generating 1,6-Anhydrosugars Consisting of Amino Monosaccharide Backbones

**DOI:** 10.3390/molecules25081944

**Published:** 2020-04-22

**Authors:** Harumi Kaga, Masaru Enomoto, Hiroki Shimizu, Izuru Nagashima, Keigo Matsuda, Seigou Kawaguchi, Atsushi Narumi

**Affiliations:** 1 National Institute of Advanced Industrial Science and Technology (AIST), Sapporo 062-8517, Japan; 2 Laboratory of Applied Bioorganic Chemistry, Graduate School of Agricultural Science, Tohoku University, 468-1 Aramaki Aza-Aoba, Aoba-ku, Sendai 980-8572, Japan; masaru.enomoto.a2@tohoku.ac.jp; 3 Bioproduction Research Institute, Department of Life Science and Biotechnology, National Institute of Advanced Industrial Science and Technology (AIST), 1-1-1 Higashi, Tsukuba, Ibaraki 305-8568, Japan; hiroki.shimizu@aist.go.jp (H.S.); izu.nagashima@aist.go.jp (I.N.); 4 Department of Chemistry and Chemical Engineering, Graduate School of Science and Engineering, Yamagata University, Jonan 4-3-16, Yonezawa 992-8510, Japan; matsuda@yz.yamagata-u.ac.jp; 5 Department of Organic Materials Science, Graduate School of Organic Materials Science, Yamagata University, Jonan 4-3-16, Yonezawa 992-8510, Japan; skawagu@yz.yamagata-u.ac.jp

**Keywords:** microwave, anhydrosugar, *N*-acetyl glucosamine (GlcNAc; NAG), anhydro-pyranose, anhydro-furanose, sulfolane, biologically active building block

## Abstract

The microwave-assisted heating reaction of *N*-acetyl glucosamine (GlcNAc) in sulfolane is described. The reaction produces two major products that are assignable to 1,6-anhydro-2-acetamido-2-deoxy-β-d-glucopyranose (AGPNAc) and 1,6-anhydro-2-acetamido-2-deoxy-β-d-glucofuranose (AGFNAc). In order to reveal a general feature of the system, the 3, 5, and 10 min reactions were performed at 140, 160, 180, 200, and 220 °C to clarify the time course changes in the conversion of GlcNAc and the yields of the two produced 1,6-anhydrosugars. Temperature is a crucial factor that significantly affects the conversion of GlcNAc. The yields of AGPNAc and AGFNAc are also drastically changed depending on the reaction conditions. The 5-min reaction at 200 °C is shown to be the optimal condition to generate the 1,6-anhydrosugars with a high efficiency in which AGPNAc and AGFNAc are produced in the yields of 21% and 44%, respectively. Consequently, the microwave-assisted heating reaction of GlcNAc in sulfolane is shown to be a simple and promising pathway to generate 1,6-anhydrosugars consisting of amino monosaccharide backbones, which have high potentials as raw materials leading to biological oligosaccharides and biomimetic polysaccharides.

## 1. Introduction

1,6-Anhydrosugars are structurally featured bicyclo molecules in which their inherent 1,6-linkages can react with nucleophiles and/or electrophiles under selected conditions; thus, they have high potentials as building blocks for biologically-potent oligosaccharides [1], monomers to prepare linear and branched polysaccharides [2,3,4,5,6,7], and characteristic raw materials to construct porous polysaccharides [8]. The microwave-assisted heating reaction of monosaccharides is one promising methodology directly affording the 1,6-anhydrosugars. As an example, we have reported the microwave-irradiated heating reactions of d-mannose and its derivatives in sulfolane, which produces 1,6-anhydro-β-d-mannopyranose (AMP) and 1,6-anhydro-β-d-mannofuranose (AMF) [9]. Similarly, methyl-α,β-d-glucofuranose transforms into 1,6-anhydro-β-d-glucofuranose (AGF) [10].

The amide derivatives of amino monosaccharides, such as *N*-acetylglucosamine (GlcNAc), *N*-acetylgalactosamine (GalNAc), and *N*-acetylmannosamine (ManNAc), are particularly crucial ones that are categorized as the “eight essential monosaccharides for humans to maintain life” [11]. Oligosaccharides composed of diverse combinations of such eight essential monosaccharides are known to play informational roles in biological systems [12]. As a well-known example, the oligosaccharides consisting of GlcNAc, GalNAc, and two other monosaccharides exist on the surface of human red blood cells to determine the ABO blood groups. Sialic acids targeted by the influenza virus contain the ManNAc backbone in their molecules. Furthermore, GlcNAc is a unit of structural biopolymers, such as chitin, hyaluronic acid, and peptidoglycans. Anhydrosugars consisting of amino monosaccharide backbones are powerful molecular tools [13] leading to such biological oligosaccharides and biomimetic polysaccharides, so the development of their facile synthetic routes is an important issue to be studied.

Indeed, several groups have previously reported the synthetic routes for such anhydrosugars. The 1,6-anhydrosugar consisting of a pyranose ring, such as 1,6-anhydro-2-acetamido-2-deoxy-β-d-glucopyranose (AGPNAc), has been prepared from *N*-benzylidene-d-glucosamine via a five-step reaction [14]. AGPNAc is also prepared from 4,6-*O*-benzylidene-β-d-glucopyranoside, while this route includes a total of 12 reactions [15]. The tosylation of the primary OH group of GlcNAc followed by the treatment with 1,8-diazabicyclo[5.4.0]undec-7-ene (DBU) has been reported to produce AGPNAc [16]. The 1,6-anhydrosugar consisting of a furanose ring, such as 1,6-anhydro-2-acetamido-2-deoxy-β-d-glucofuranose (AGFNAc), is another related anhydrosugar. AGFNAc has been suggested to exist in smoke from the charring/burning of chitin [17], whereas neither its isolation nor assignment has been achieved.

**Scheme 1:**
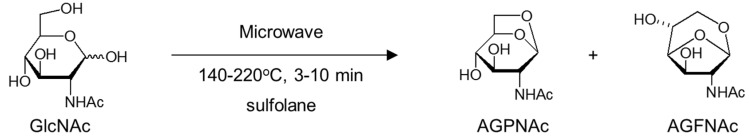
The microwave-assisted heating reaction of *N*-acetyl glucosamine (GlcNAc) producing anhydrosugars, such as 1,6-anhydro-2-acetamido-2-deoxy-β-d-glucopyranose (AGPNAc) and 1,6-anhydro-2-acetamido-2-deoxy-β-d-glucofuranose (AGFNAc).

In this study, we report the microwave-assisted heating reaction of *N*-acetyl glucosamine (GlcNAc) as shown in Scheme 1. We proved that this reaction system is a facile one producing two types of 1,6-anhydro amino sugars in the forms of the pyranose ring and/or furanose ring, such as AGPNAc and AGFNAc. In particular, AGFNAc has been for the first time isolated and fully assigned. The time course changes in the conversion of GlcNAc and the yields of the two produced 1,6-anhydrosugars, AGPNAc and AGFNAc, were evaluated, which are significantly dependent on the reaction temperature within the short reaction times up to 10 min.

## 2. Results and Discussion

### 2.1. Products Assignments

**Chart 1:**
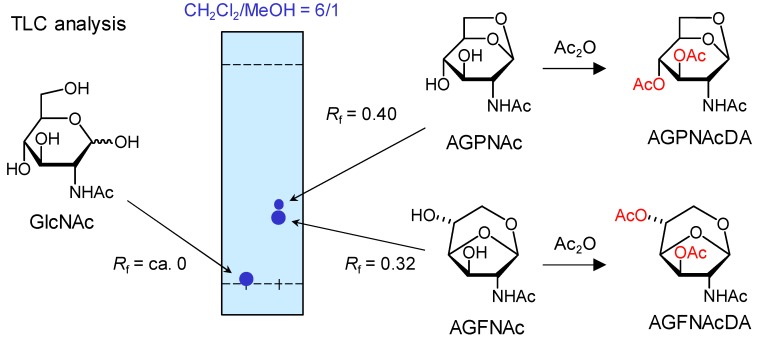
Image for the results of the TLC analyses to explain the reaction features and scheme for acetylation to ensure the molecular assignments.

The microwave-assisted heating reaction of *N*-acetyl glucosamine (GlcNAc) was initially performed in sulfolane as the solvent at 220 °C for 3 min to obtain a reaction product. Chart 1 shows an image for the results of the thin-layer chromatography (TLC) analyses using CH_2_Cl_2_/MeOH = 6/1 as an eluent together with the *R*_f_ values of the compounds. The spot due to GlcNAc appears at *R*_f_ = ca. 0, which almost disappears after the reaction. Hence, GlcNAc has been consumed in a high conversion. Alternatively, the spots after the reaction include new ones at *R*_f_ = 0.32 and *R*_f_ = 0.40, suggesting that the compounds with less polarity are produced by the microwave-assisted reaction. We could observe only three spots due to the residual starting material (GlcNAc) and reaction products (AGPNAc and AGFNAc) for the TLC analysis. The respective products have been isolated by the silica gel column chromatography. The less polar product with *R*_f_ = 0.40 is assignable to 1,6-anhydro-2-acetamido-2-deoxy-β-d-glucopyranose (AGPNAc) because its chemical data is in accordance with AGPNAc that was prepared by the conventional chemical method [14]. We performed acetylation for AGPNAc because it is known to be a convenient method to provide a clear proof for molecular assignments. The chemical data for the acetylation product fairly agrees with that of 1,6-anhydro-2-acetamido-2-deoxy-3,4-*O*-diacetyl-β-d-glucopyranose (AGPNAcDA) reported in the literature [14,18]. These results again support the fact that the reaction has produced AGPNAc.

**Chart 2:**
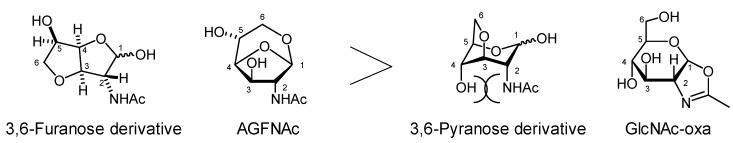
Structural candidates for the product with *R*_f_ = 0.32.

Next, we focused on the assignment of the more polar product with *R*_f_ = 0.32 (Chart 1). The high-resolution mass spectra (HRMS/ESI) and elementary analyses indicated that the formula of the product perfectly agreed with that of AGPNAc (C_8_H_13_NO_5_). Hence, we propose four isomers of AGPNAc including 3,6-anhydro-2-acetamido-2-deoxy-β-d-glucofuranose (3,6-furanose derivative), 1,6-anhydro-2-acetamido-2-deoxy-β-d-glucofuranose (AGFNAc), 3,6-anhydro-2-acetamido-2-deoxy-β-d-glucopyranose (3,6-pyranose derivative), and GlcNAc-oxa as structural candidates for the product with *R*_f_ = 0.32 (Chart 2). Anomeric mixtures of the 3,6-furanose derivative have been previously reported by Ogata et al. [19], while their chemical and physical data are significantly different from those of the obtained product. For the 3,6-pyranose derivative, the steric hindrance due to the diaxial interaction between the OH group and the NHAc group is very high so that the structure is not thermodynamically preferred. We have modified the more polar product into the acetylated compound (Chart 1) in order to provide clear insights into the structure of the original product, as already described. In this case, we can determine the number of hydroxyl groups in the original compound by this derivatization. The ^1^H NMR measurements for the acetylation product indicated that two acetyl groups were incorporated so that the original product possesses two OH groups. Thus, GlcNAc-oxa bearing three OH is excluded from the possible candidates for the major product with *R*_f_ = 0.32. On the other hand, all signals observed in both the ^1^H- and ^13^C NMR spectra of the product can be reasonably assignable to that of AGFNAc. Furthermore, the acetylated product is fully assigned to 1,6-anhydro-2-acetamido-2-deoxy-3,5-*O*-diacetyl-β-d-glucofuranose (AGFNAcDA) (Chart 1). Thus, we concluded that the microwave-assisted 3-min heating reaction of GlcNAc at 220 °C in sulfolane is a promising condition to produce AGPNAc and AGFNAc, as shown in Scheme 1. Other plausible reaction products include oligomers and low molecular weight compounds analogous to those obtained by high-temperature steam treatment [20]. Noteworthy, is that the formation of AGFNAc is for the first time, experimentally proven.

### 2.2. Reaction System Clarification

**Figure 1:**
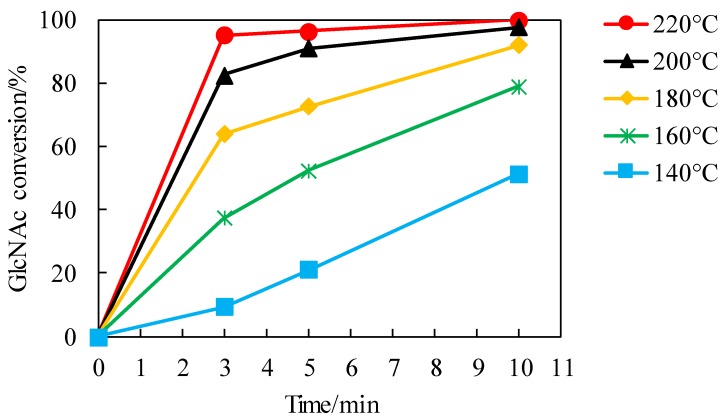
The plots of the GlcNAc conversion as a function of the reaction time.

In order to clarify the general features for the microwave-assisted heating reactions of GlcNAc in sulfolane, we have performed the reactions at various temperatures, such as 140, 160, 180, 200, and 220 °C. An aliquot of the reaction mixture was injected into the HPLC system and the conversions of GlcNAc were quantified by the area integration of the peak in the retention time (RT) = 16.1 min. Figure 1 shows a plot of the GlcNAc conversion as a function of the reaction time, indicating that temperature is a crucial factor that significantly affects the conversion of GlcNAc. For example, the conversions after 3 min were 9%, 38%, 64%, 83%, and 95% at the respective temperatures. In general, the conversions increase with the increasing reaction times.

**Figure 2:**
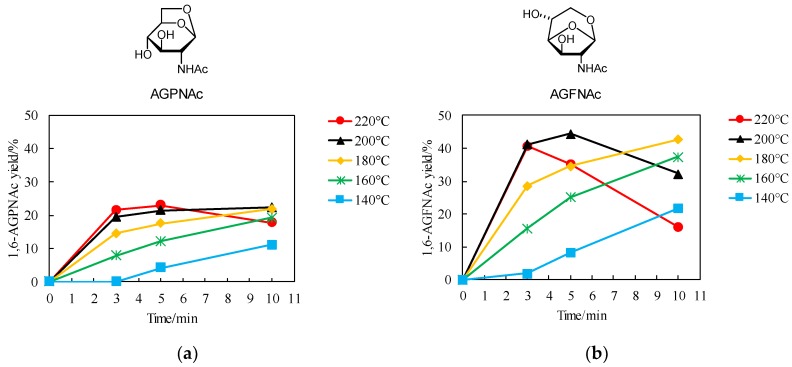
The plots of the yields of (**a**) AGPNAc and (**b**) AGFNAc as a function of the reaction time.

The yield of AGPNAc has been determined by the area integration for the peak at RT = 17.9 min for the HPLC measurements. Figure 2a shows the yields of AGPNAc as a function of the reaction time. The yields drastically changed depending on the selected reaction conditions. For example, the yields after 3 min were 0%, 7.9%, 15%, 20%, and 22% at 140, 160, 180, 200, and 220 °C, respectively, which slightly increased to 4.2%, 12%, 17%, 21%, and 23% after 5 min, respectively. In general, the higher temperature is preferred for the formation of AGPNAc. The highest value of 23% was obtained at 5 min and 220 °C. For the 220 °C system, the yield was reduced to 18% after 10 min. A possible reason is the occurrence of the decomposition of AGPNAc at this temperature, which is supported by the result that AGPNAc decomposes at 207 °C [14].

The yields of AGFNAc have been similarly determined by the area integrations for the peak at RT = 18.9 min for the HPLC measurements and plotted in Figure 2b. The 140, 160, and 180 °C systems are first described. A featured tendency is that AGFNAc (Figure 2b) is preferentially produced versus AGPNAc (Figure 2a). A possible reason is discussed in the next section. The yields of AGFNAc were greatly dependent on the reaction conditions. The yields after 3 min were 1.9%, 16%, and 28% at 140, 160, and 180 °C, respectively, which increased to 8.3%, 25%, and 35%, respectively, after 5 min (Figure 2b). The respective yields increased to 22%, 37%, and 43% after 10 min, respectively. The yields increased with the increasing reaction times. The 10-min reaction at 180 °C was concluded to be the optimal condition to produce AGFNAc in the high yield of 43%. On the other hand, for the 200 and 220 °C systems, the yields decreased by increasing the reaction times. For example, the yield reached 44% at 5 min for the 200 °C system, which decreased to 32% at 10 min. The decrease is noticeable for the 220 °C system in which the yields were 41% at 3 min, 35% at 5 min, and 16% at 10 min. These results suggest that AGFNAc is thermodynamically unstable as compared with AGPNAc.

### 2.3. Selectivity to Form Pyranose Ring Versus Furanose Ring Differed from other Systems

The microwave-assisted heating reaction of GlcNAc is shown to generate the corresponding anhydro amino sugars, such as AGPNAc and AGFNAc. Furthermore, we have, for the first time, proven that the furanose selectivity is high for this system, as already described. Notably, this tendency is considerably different from those obtained for the microwave reaction systems using mannose (Man) and glucose (Glc) as the starting materials. For example, 1,6-anhydro-pyranose is predominantly formed for the Man system [9]. Similarly, for the systems using Glc and/or Glc-repeating polymers as the starting materials, the selectivity for the formation of the 1,6-anhydro-pyranose derivative is higher than that of the 1,6-anhydro-furanose one by the thermochemical transformation of Glc in high-temperature steam [20], microwave heating of Glc in an ionic liquid [21], thermal degradation of cellulose in supercritical acetone [22], and microwave pyrolysis of cellulosic materials [23,24].

**Chart 3:**
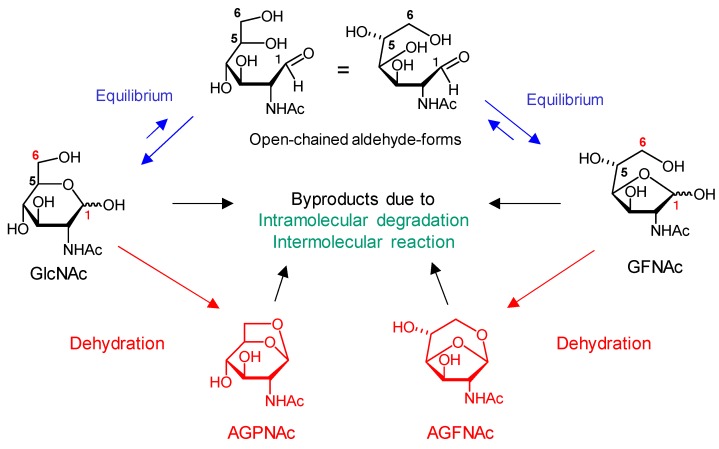
A mechanistic explanation involved in the microwave-assisted heating reaction of GlcNAc in sulfolane.

Chart 3 presents the possible pathways for the microwave-assisted heating reaction of GlcNAc. At the relatively high temperatures of 140–220 °C in a sulfolane solution, the intramolecular transformation of GlcNAc into the furanose form (GFNAc) may be allowed to occur through its open-chained aldehyde forms, which are similar to other monosaccharide systems. The intramolecular dehydration reaction of the formed GFNAc is a possible way to produce AGFNAc. This speculation seems to be rational as we consider the following facts. Certain sugars can exist as a furanose form to a greater extent in dimethyl sulfoxide (DMSO) than in water [25,26]. The furanose form/pyranose form ratio tends to increase with the increasing temperature [27]. These reports are similar to the conditions of this study, in that the reactions have been conducted at the relatively high temperatures of 140–220 °C in sulfolane. It should be stated that sulfolane is a solvent that is structurally similar to DMSO. Furthermore, there is a report on the transformation of GlcNAc into 3-acetamido-5-acetylfuran at temperatures of 160–210 °C in which the formation of the five-membered ring intermediate is shown to be key [28,29]. Hence, there is a possibility that the furanose form (GFNAc) exists more than the pyranose form (GlcNAc). An additional speculation is that the reaction constant for the intramolecular dehydration reaction from GFNAc to AGFNAc is higher than that from GlcNAc to AGPNAc. For such a case, once GFNAc is formed, it will readily transform into AGFNAc. Eventually, AGFNAc can be preferentially formed even if GFNAc exists less than GlcNAc. This assumption implies that the activation energy for the dehydration reaction of GFNAc to AGFNAc might be lower than that of GlcNAc to AGPNAc. Consequently, the 1,6-anhydrosugars consisting of amino monosaccharide backbones, such as AGPNAc and AGFNAc, are produced via a very simple reaction with a good reproducibility. At least, GlcNAc (starting material), GFNAc (intermediate), and AGPNAc/AGFNAc (products) are the key monomeric species of this system. For the respective species, an intramolecular degradation, such as a ring-opening reaction and bond-cleavage, together with an intermolecular reaction, such as dimerizations and oligomerizations, possibly occur, eventually providing byproducts, since similar reactions have been reported to proceed between the 1,6-anhydrosugars [9,20]. As with the TLC analysis, the HPLC trace exhibited three peaks corresponding to GlcNAc, AGPNAc, and AGFNAc. No typical peaks were observed except for those three. Hence, the structures of the formed byproducts seem to be diverse and difficult to be assigned. In this study, however, the reaction to generate the target AGPNAc and AGFNAc has been optimized. An establishment of a facile preparative protocol of such anhydrosugars provides opportunities to study a new class of biologically-potent oligosaccharides and structurally bio-mimic polymers and/or non-natural linear and branched polysaccharides.

## 3. Materials and Methods

### 3.1. Materials

*N*-Acetyl glucosamine (GlcNAc, >95%, Wako Pure Chemical Industries, Osaka, Japan) was recrystallized from methanol. Tetramethylene sulfone (sulfolane, >99%) and tetraethylene glycol dimethyl ether (TEGDME, >99%) were supplied from Sigma-Aldrich Japan (Tokyo, Japan). Acetic anhydride (>97.0%), dimethylaminopyridine (DMAP, >99.0%), pyridine (>99.0%), and DMSO (>99.0%) were obtained from Wako Pure Chemical Industries (Osaka, Japan). The solvents and commercially-available reagents were used as received without further purification unless otherwise stated. Sulfolane was liquefied with 2% TEGDME to make its handling easier. Column chromatography and thin layer chromatography (TLC) were performed using silica gel, Kanto Chemicals (Tokyo, Japan) 60N (sphere) No. 37565-84 and Merck Art. 5715, respectively.

### 3.2. Instruments

The microwave-assisted heating was carried out using a Green Motif-I apparatus (IDX Co, Ltd., Sano, Japan; single mode; output power 30–300 W) at 2.45 GHz [9]. The ^1^H and ^13^C NMR (400 MHz and 100 MHz, respectively) spectra were measured using a JNM-ECP400 spectrometer (JEOL Ltd., Tokyo, Japan) in D_2_O or CDCl_3_ with DSS or Me_4_Si as the internal standard (δ = 0 ppm). Optical rotations were measured by a DIP-360 digital polarimeter (JASCO Corporation, Tokyo, Japan). Melting points were determined using a 500-D micromelting point apparatus (Yanaco Analytical Systems Inc., Kyoto, Japan) and were uncorrected. High-resolution mass spectra (HRMS/ESI) data were obtained by a Thermo Scientific Exactive (Thermo Fisher Scientific K. K., Tokyo, Japan). Elemental analysis was conducted using a JM10 CHN Analyzer MICRO CORDER (J-Science Lab Co., Ltd., Kyoto, Japan). The high-performance liquid chromatography (HPLC) was performed using a Gilson HPLC system (Gilson Incorporated, Wisconsin, WI, USA) with a Shodex KS-801 column [80 °C, H_2_O, 0.5 mL/min] and SEDEX 55 ELSD detector.

### 3.3. Microwave-Assisted Heating Reaction and HPLC Analysis

To the starting material (GlcNAc, 100 mg) in a 20 mL test tube, liquefied sulfolane (5.0 mL) was added. Further details about the microwave-assisted heating reaction have been reported elsewhere [9]. The reaction mixture was diluted with DMSO (1.0 mL). An aliquot (0.3 mL) of the mixture was added to H_2_O (3.0 mL). The resultant mixture was subjected to HPLC.

### 3.4. Isolation of 1,6-Anhydro-2-acetamido-2-deoxy-β-d-glucopyranose (AGPNAc) and 1,6-Anhydro-2-acetamido-2-deoxy-β-d-glucofuranose (AGFNAc)

Microwave-assisted heating of GlcNAc (100 mg) in liquefied sulfolane (5 mL) was repeated 4 times at 220 °C for a 5-min irradiation time. The combined reaction mixtures were then directly purified twice by column chromatography on silica gel (60 mL, eluted with 5% MeOH/CH_2_Cl_2_, then 9% MeOH/CH_2_Cl_2_) to give 1,6-anhydro-2-acetamido-2-deoxy-β-d-glucopyranose (AGPNAc) (59.6 mg, 16.2%) and 1,6-anhydro-2-acetamido-2-deoxy-β-d-glucofuranose (AGFNAc) (136 mg, 37.1%) as colorless solids. AGPNAc and AGFNAc were crystallized from *i*-PrOH/AcOEt and MeOH/AcOEt, respectively, to give the samples for the analyses. Data for AGPNAc: mp 190–191 °C, *R*_f_ = 0.40 (CH_2_Cl_2_/MeOH 6:1), [α]_D_^25^ − 46.1° (c 1.01, H_2_O); lit. [14] mp 190–191 °C, [α]_D_^20^ − 45.2° (c 2.3, H_2_O); lit. [17] mp 189–191 °C, [α]_D_^20^ − 42.5° (c 2.2, H_2_O); lit. [18] mp 193 °C, [α]_D_^20^ − 45° (c 1, MeOH); ^1^H NMR (400 MHz, D_2_O): δ 5.43 (s, 1H, H-1), 4.64 (d, *J* = 5.4, H-5), 4.18 (dd, *J* = 7.8, 1.0, H-6b), 3.73–3.80 (3H, m, H-2, H-3, H-6a), 3.65 (m, H-4), 2.03 (s, 3H); ^13^C NMR (100 MHz, D_2_O): δ 176.5 (NC=O), 103.5 (C-1), 78.8 (C-5), 73.7 (C-3), 73.3 (C-4), 68.1 (C-6), 54.8 (C-2), 24.6 (CH_3_CON); HRMS (ESI): *m*/*z* calcd for C_8_H_13_NO_5_Na ([M + Na]^+^) 226.0686, found 226.0686; Anal. calcd for C_8_H_13_NO_5_: C, 47.29; H, 6.45; N, 6.89. Found C, 47.11; H, 6.41; N, 6.79. Data for AGFNAc: mp: 197–198 °C; *R*_f_ = 0.32 (CH_2_Cl_2_/MeOH 6:1); [α]_D_^25^ + 41.6° (c 1.01, H_2_O); ^1^H NMR (400 MHz, D_2_O): δ 5.10 (s, 1H, H-1), 4.40–4.42 (m, 1H, H-2), 4.42–4.45 (m, 2H, H-4, and H-5), 4.36 (dd, 1H, *J* = 13.5, 2.7, H-6b), 3.86 (m, 1H, H-3), 3.86 (d, 1H, *J* = 13.5, H-6a), 2.02 (s, 3H); ^13^C NMR (100 MHz, D_2_O): δ 176.9 (NC=O), 105.7 (C-1), 82.4 (C-5), 78.4 (C-3), 68.6 (C-4), 66.2 (C-6), 62.9 (C-2), 24.4 (CH_3_CON); HRMS (ESI): *m*/*z* calcd for C_8_H_13_NO_5_Na ([M + Na]^+^), 226.0686, found 226.0685; Anal. calcd for C_8_H_13_NO_5_: C, 47.29; H, 6.45; N, 6.89. Found C, 47.08; H, 6.61; N, 6.91. (Supplementary Materials Figure S1.1–S1.3 and Figure S2.1–S2.3).

### 3.5. Acetylation of AGPNAc

Acetic anhydride (0.12 mL, 1.28 mmol) was added to a suspension of AGPNAc (45.8 mg, 0.23 mmol) in ethyl acetate (4 mL), a catalytic amount of DMAP, and pyridine (0.12 mL, 1.49 mmol). After stirring at room temperature for 1 h, an ethanol (0.2 mL) solution in ethyl acetate (2 mL) was added to the mixture, followed by NaHCO_3_ (168 mg, 2.00 mmol). The mixture was then directly purified by short column chromatography on silica gel (10 mL, eluted with AcOEt/hexane 2:1) to give 1,6-anhydro-2-acetamido-2-deoxy-3,4-*O*-diacetyl-β-d-glucopyranose (AGPNAcDA) (65 mg, quantitatively). AGPNAcDA was crystallized from AcOEt/hexane to give a sample for the analysis. Data for AGPNAcDA: mp 138.5–139.5 °C, [α]_D_^25^ − 95.5° (c 1.12, CHCl_3_); lit. [14] mp 137–138 °C, [α]_D_^20^ − 88.4° (c 1.1, MeOH); lit. [18] mp 139 °C, [α]_D_^20^ − 97° (c 1, CHCl_3_); ^1^H NMR (400 MHz, CDCl_3_): δ 5.93(d, 1H, *J* = 9.5, NH), 5.38 (s, 1H, H-1), 4.73 (m, 1H, H-3), 4.67 (m, 1H, H-4), 4.60 (d, *J* = 5.1, 1H, H-5), 4.16 (d, 1H, *J* = 7.8, H-6b), 4.11 (d, 1H, *J* = 9.7, H-2), 3.83 (dd, 1H, *J* = 7.6, 5.7, H-6a), 2.18 and 2.12 (each s, each 3H, AcO), 2.04 (s, 3H, AcN); ^13^C NMR (100 MHz, CDCl_3_): δ 169.30 and 169.30 (each OC=O), 169.0 (NC=O), 100.8 (C-1), 73.5 (C-5), 70.2 (C-3), 70.1 (C-4), 65.4 (C-6), 49.1 (C-2), 23.2 (CH_3_CON), 20.95 and 20.9 (2 × CH_3_COO); HRMS (ESI): *m*/*z* calcd for C_12_H_17_NO_7_Na ([M + Na]^+^), 310.0897, found 310.0897; Anal. calcd for C_12_H_17_NO_7_: C, 50.17; H, 5.96; N, 4.88. Found C, 50.10; H, 6.10; N, 4.87. (Supplementary Materials Figure S3.1–S3.3).

### 3.6. Acetylation of AGFNAc

The same procedure as that for the acetylation of AGPNAc was applied to AGFNAc (100 mg, 0.492 mmol) to give 1,6-anhydro-2-acetamido-2-deoxy-3,5-*O*-diacetyl-β-d-glucofuranose (AGFNAcDA) (141 mg, quantitatively) as a colorless syrup. Data for AGFNAcDA: [α]_D_^25^ − 11.1° (c 1.01, CHCl_3_); ^1^H NMR (400 MHz, CDCl_3_): δ 6.22 (d, 1H, *J* = 7.0, NH), 5.16 (s, 1H, H-1), 5.11 (dd, 1H, *J* = 7.0, 3.2, H-5), 4.7 (m, 1H, H-4), 4.64 (d, 1H, *J* = 6.8, H-2), 4.63 (d, 1H, *J* = 7.2, H-3), 4.33 (dd, 1H, *J* = 13.5, 2.7, H-6b), 3.96 (d, 1H, *J* = 13.5, H-6a), 2.19 and 2.17 (each s, each 3H, AcO), 2.04 (s, 3H, AcN); ^13^C NMR (100 MHz, CDCl_3_): δ 170.6 (NC=O), 170.2 and 170.1 (each OC=O), 102.9 (C-1), 77.4 (C-5), 75.9 (C-4), 66.1 (C-3), 63.2 (C-6), 58.3 (C-2), 22.9 (CH_3_CON), 21.1 and 20.6 (2× CH_3_COO); HRMS (ESI): *m*/*z* calcd for C_12_H_17_NO_7_Na ([M + Na]^+^), 310.0897, found 310.0897. (Supplementary Materials Figure S4.1–S4.3).

## 4. Conclusions

The microwave-assisted heating reaction of *N*-acetyl glucosamine (GlcNAc) in sulfolane has been demonstrated, which is shown to be a facile methodology to produce two types of 1,6-anhydro amino sugars in the forms of the pyranose ring and/or furanose ring, such as 1,6-anhydro-2-acetamido-2-deoxy-β-d-glucopyranose (AGPNAc) and 1,6-anhydro-2-acetamido-2-deoxy-β-d-glucofuranose (AGFNAc). In particular, AGFNAc has been, for the first time, synthesized and fully assigned. The yields of AGPNAc and AGFNAc are significantly dependent on the reaction temperature within the short reaction times up to 10 min. The 5 min reaction at 200 °C was shown to be one efficient condition to transform GlcNAc (91% conversion) into AGPNAc (21% yield) and AGFNAc (44% yield). This simple method with a good reproducibility integrates the use of 1,6-anhydrosugars consisting of amino monosaccharide backbones.

## Supplementary Materials

The following are available online, Figure S1.1: ^1^H NMR spectrum of AGPNAc (400 MHz, D_2_O), Figure S1.2: ^13^C NMR spectrum of AGPNAc (100 MHz, D_2_O), Figure S1.3: ESI-HRMS of AGPNAc along with analytical data, Figure S2.1: ^1^H NMR spectrum of AGFNAc (400 MHz, D_2_O), Figure S2.2: ^13^C NMR spectrum of AGFNAc (100 MHz, D_2_O), Figure S2.3: ESI-HRMS of AGFNAc along with analytical data, Figure S3.1: ^1^H NMR spectrum of AGPNAcDA (400 MHz, CDCl_3_), Figure S3.2: ^13^C NMR spectrum of AGPNAcDA (100 MHz, CDCl_3_), Figure S3.3: ESI-HRMS of AGPNAcDA along with analytical data, Figure S4.1: ^1^H NMR spectrum of AGFNAcDA (400 MHz, CDCl_3_), Figure S4.2: ^13^C NMR spectrum of AGFNAcDA (100 MHz, CDCl_3_), Figure S4.3: ESI-HRMS of AGFNAcDA along with analytical data.
