# Supplementary material for: Microwave-Assisted Heating Reactions of N-Acetylglucosamine (GlcNAc) in Sulfolane as a Method Generating 1,6-Anhydrosugars Consisting of Amino Monosaccharide Backbones
Source: Molecules. 2020 Apr 22;25(8):1944. doi: 10.3390/molecules25081944 (PMC7221957; doi:10.3390/molecules25081944)
Supplement: Supplementary file 1 [file molecules-25-01944-s001.pdf]

Supporting Information

Microwave-assisted heating reactions of *N*-acetylglucosamine (GlcNAc) in sulfolane as a method generating 1,6-anhydrosugars consisting of amino monosaccharide backbones

Harumi Kaga [1,*], Masaru Enomoto [2], Hiroki Shimizu [3], Izuru Nagashima [3], Keigo Matsuda [4], Seigou Kawaguchi [5] and Atsushi Narumi [5,*]

[1] National Institute of Advanced Industrial Science and Technology (AIST); Sapporo 062-8517, Japan; h.kaga@aist.go.jp
[2] Laboratory of Applied Bioorganic Chemistry, Graduate School of Agricultural Science, Tohoku University; 468-1 Aramaki Aza-Aoba, Aoba-ku, Sendai 980-8572, Japan; masaru.enomoto.a2@tohoku.ac.jp
[3] Bioproduction Research Institute, Department of Life Science and Biotechnology, National Institute of Advanced Industrial Science and Technology (AIST); 1-1-1 Higashi, Tsukuba, Ibaraki, 305-8568 Japan; hiroki.shimizu@aist.go.jp, izu.nagashima@aist.go.jp
[4] Department of Chemistry and Chemical Engineering, Graduate School of Science and Engineering, Yamagata University; Jonan 4-3-16, Yonezawa 992-8510, Japan; matsuda@yz.yamagata-u.ac.jp
[5] Department of Organic Materials Science, Graduate School of Organic Materials Science, Yamagata University; Jonan 4-3-16, Yonezawa 992-8510, Japan; skawagu@yz.yamagata-u.ac.jp, narumi@yz.yamagata-u.ac.jp
* Correspondence: h.kaga@aist.go.jp; Tel.: +81-11-857-8921 (H.K.) and narumi@yz.yamagata-u.ac.jp; Tel.: +81-238-26-3829 (A.N.)

Contents

1. Supporting data for AGPNAc 2
   Figure S1.1: ^{1}H NMR spectrum of AGPNAc (400 MHz, D_2O). 2
   Figure S1.2: ^{13}C NMR spectrum of AGPNAc (100 MHz, D_2O). 3
   Figure S1.3: ESI-HRMS of AGPNAc along with analytical data. 4
2. Supporting data for AGFNAc 5
   Figure S2.1: ^{1}H NMR spectrum of AGFNAc (400 MHz, D_2O). 5
   Figure S2.2: ^{13}C NMR spectrum of AGFNAc (100 MHz, D_2O). 6
   Figure S2.3: ESI-HRMS of AGFNAc along with analytical data. 7
3. Supporting data for AGPNAcDA 8
   Figure S3.1: ^{1}H NMR spectrum of AGPNAcDA (400 MHz, $CDCl_3$). 8
   Figure S3.2: ^{13}C NMR spectrum of AGPNAcDA (100 MHz, $CDCl_3$). 9
   Figure S3.3: ESI-HRMS of AGPNAcDA along with analytical data. 10
4. Supporting data for AGFNAcDA 11
   Figure S4.1: ^{1}H NMR spectrum of AGFNAcDA (400 MHz, $CDCl_3$). 11
   Figure S4.2: ^{13}C NMR spectrum of AGFNAcDA (100 MHz, $CDCl_3$). 12
   Figure S4.3: ESI-HRMS of AGFNAcDA along with analytical data. 13

1. Supporting data for AGPNAc

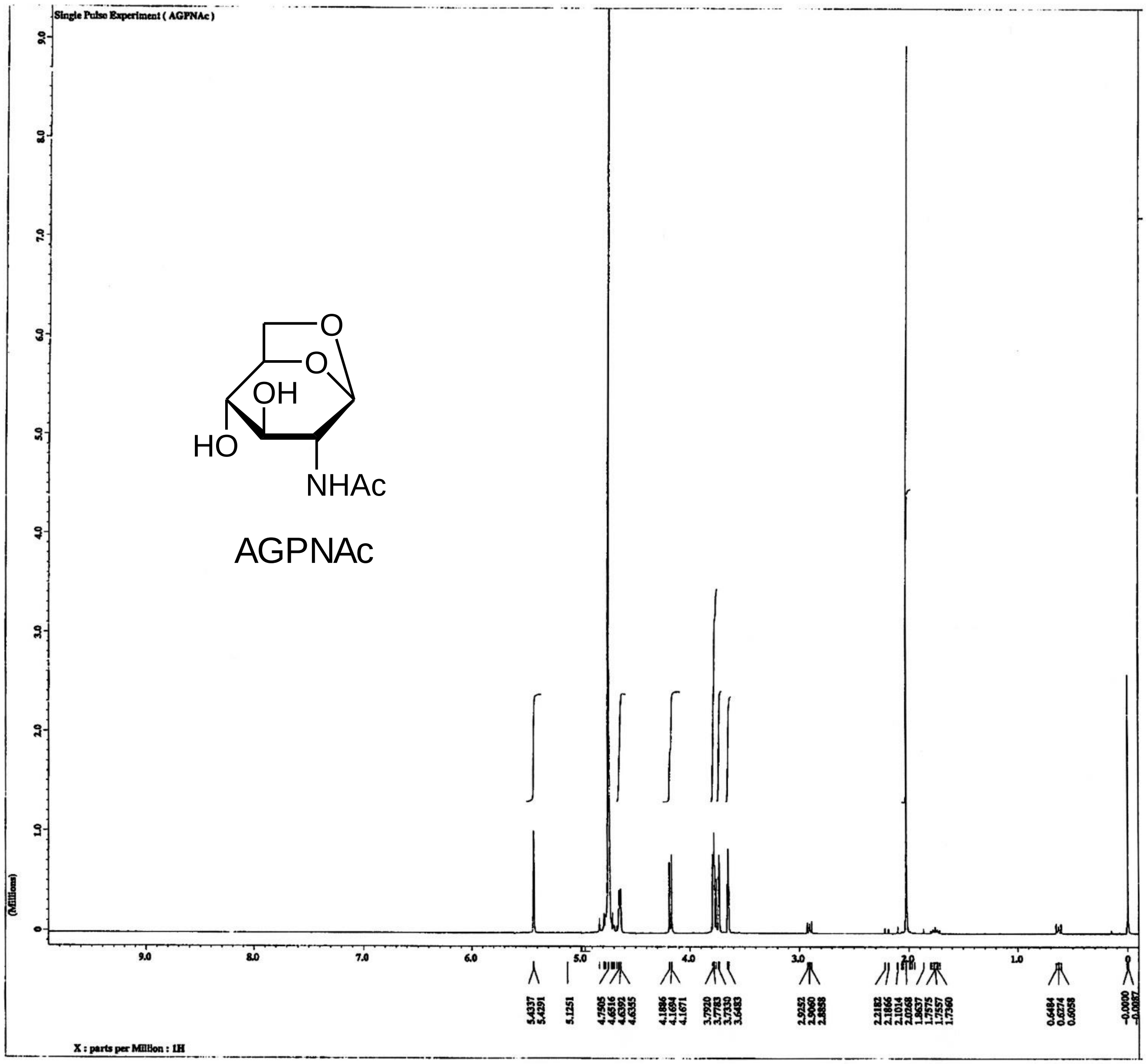

Figure S1.1: ^{1}H NMR spectrum of AGPNAc (400 MHz, D_2O).

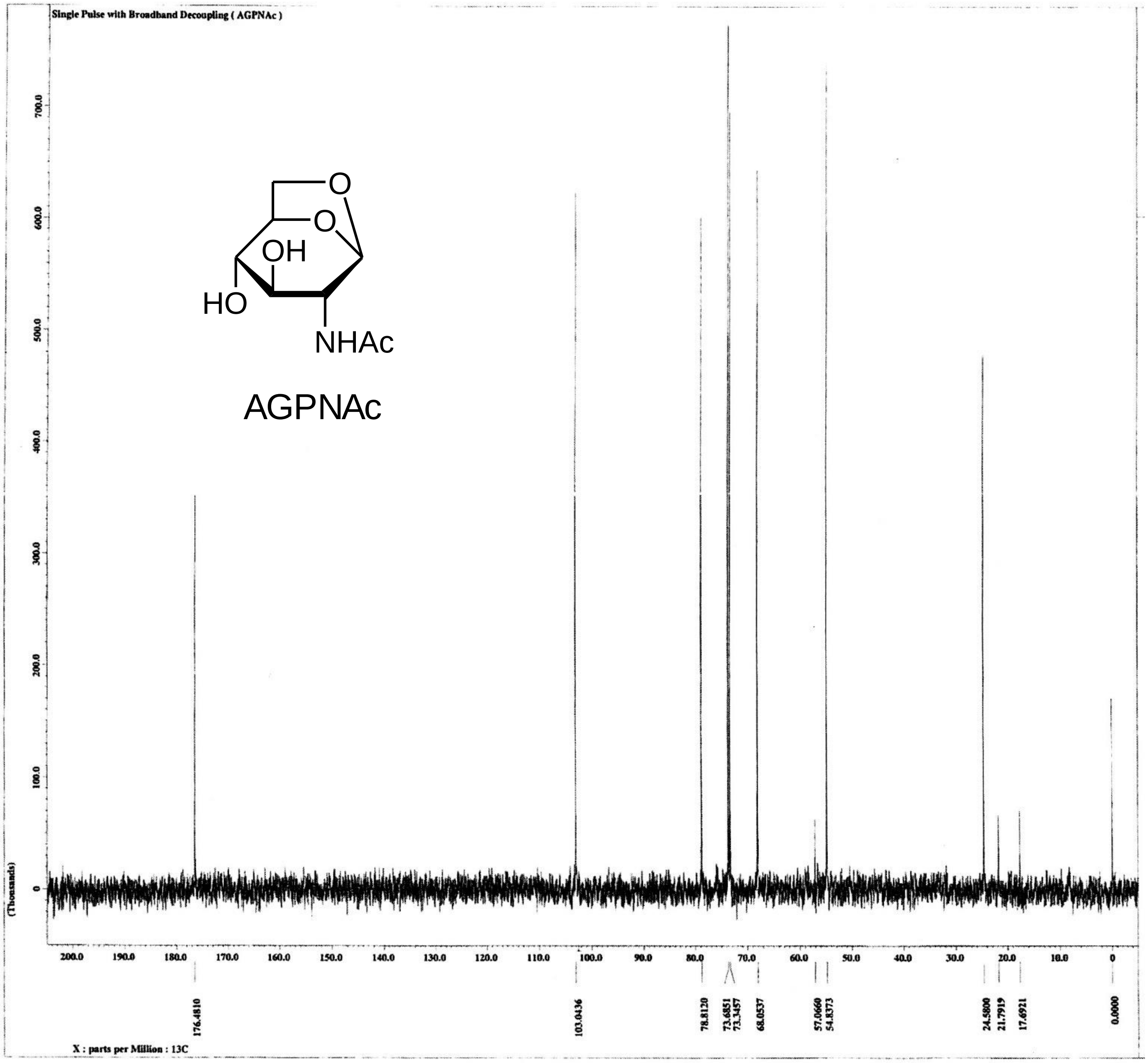

Figure S1.2: ^{13}C NMR spectrum of AGPNAc (100 MHz, D_2O).

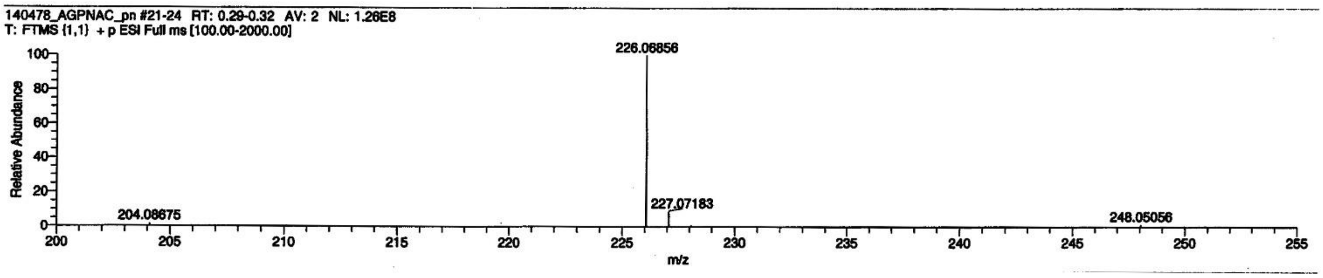

Elemental composition search on mass 226.06856

m/z= 221.06856-231.06856

Isotope	Min	Max
O-16	0	6
C-12	0	30
H-1	0	60
Na-23	0	1
N-14	0	1

Charge 1
Mass tolerance 5.00 ppm
Nitrogen rule not used
RDB equiv -1.00-100.00
max results 100

m/z	Theo. Mass	Delta (ppm)	RDB equiv.	Composition
226.06856	226.06859	-0.15	2.5	$C_8 H_{13} O_5 N Na$

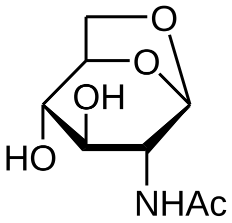

AGPNAc

Figure S1.3: ESI-HRMS of AGPNAc along with analytical data.

2. Supporting data for AGFNAc

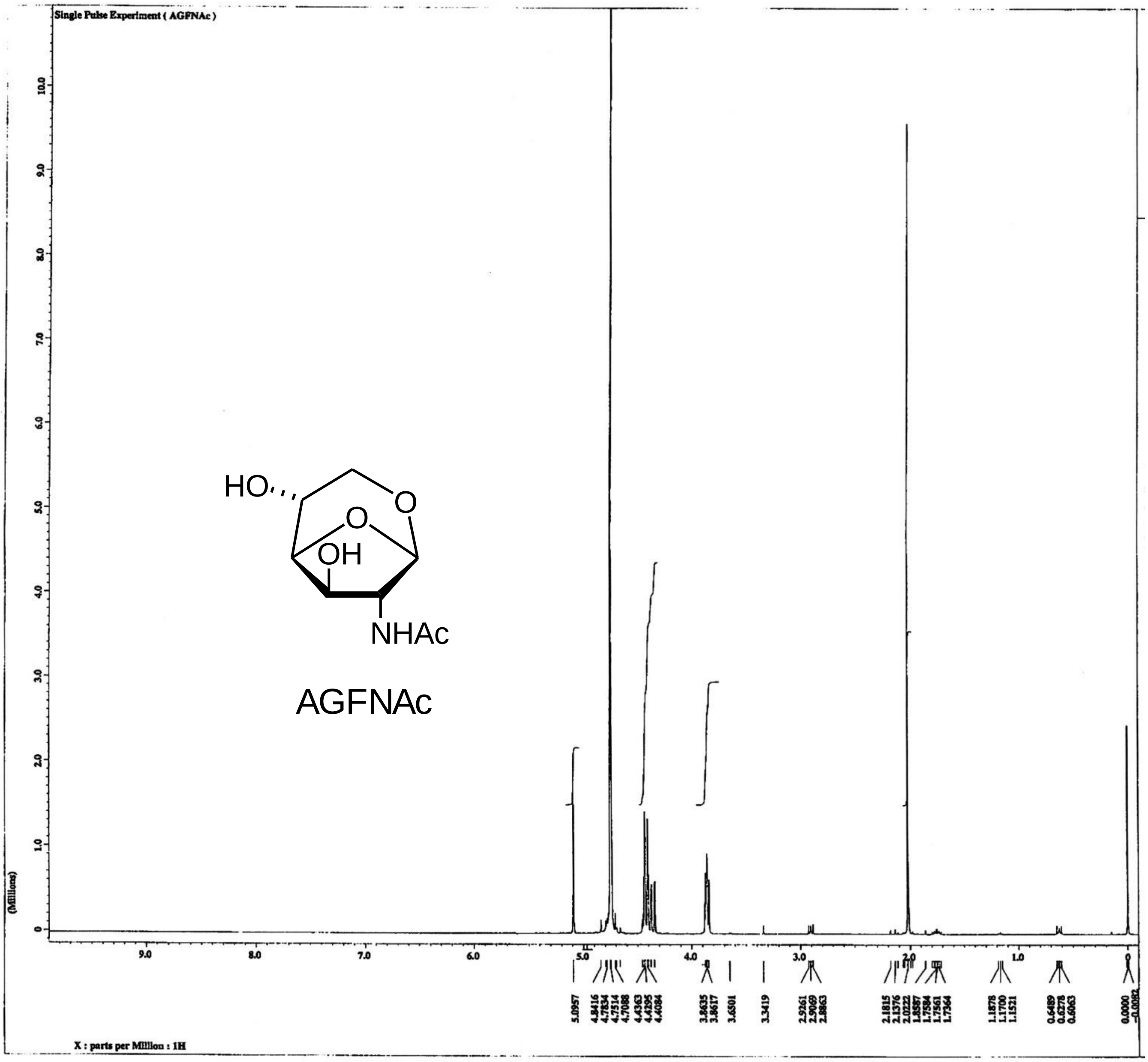

Figure S2.1: ^{1}H NMR spectrum of AGFNAc (400 MHz, D_2O).

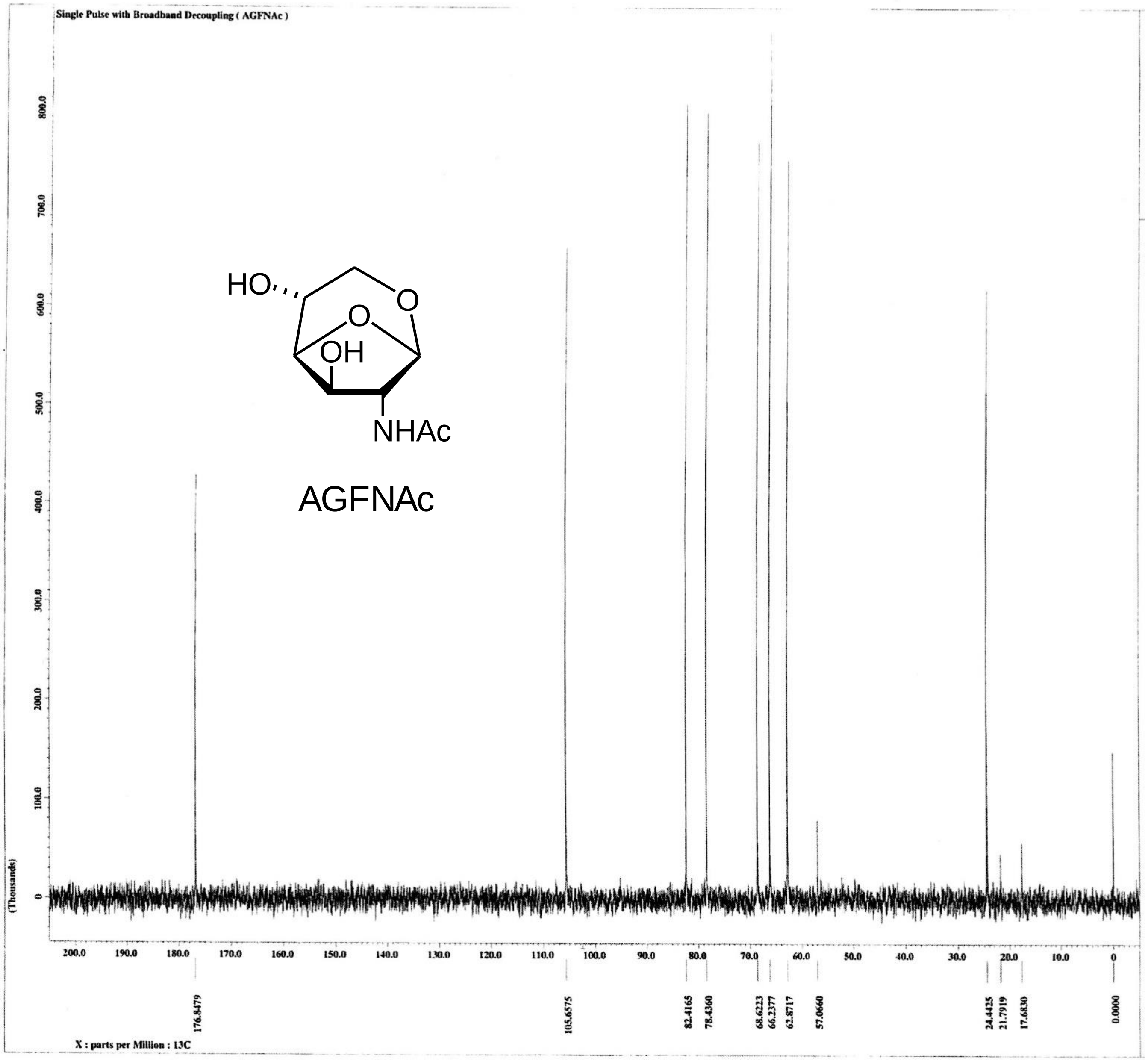

Figure S2.2: ^{13}C NMR spectrum of AGFNAc (100 MHz, D_2O).

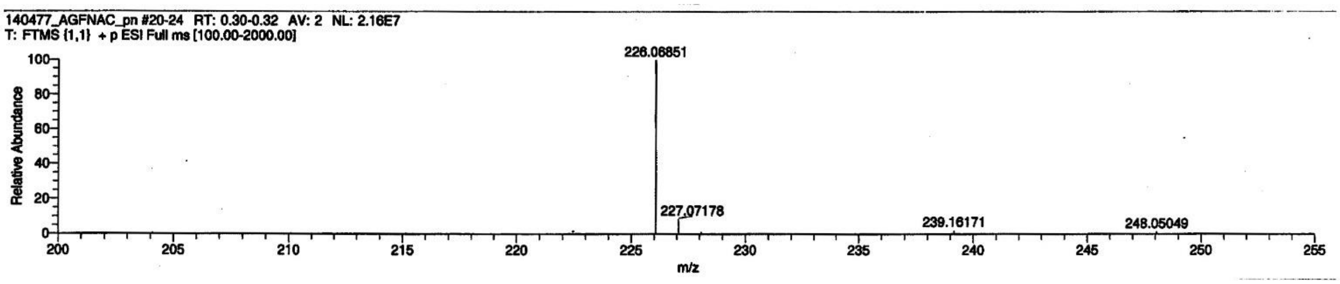

Elemental composition search on mass 226.06851

m/z= 221.06851-231.06851

Isotope	Min	Max
O-16	0	6
C-12	0	30
H-1	0	60
Na-23	0	1
N-14	0	1

Charge 1
Mass tolerance 5.00 ppm
Nitrogen rule not used
RDB equiv -1.00-100.00
max results 100

m/z	Theo Mass	Delta (ppm)	RDB equiv.	Composition
226.06851	226.06859	-0.37	2.5	$C_8 H_{13} O_5 N Na$

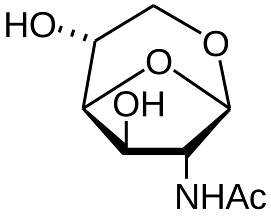

AGFNAc

Figure S2.3: ESI-HRMS of AGFNAc along with analytical data.

3. Supporting data for AGPNAcDA

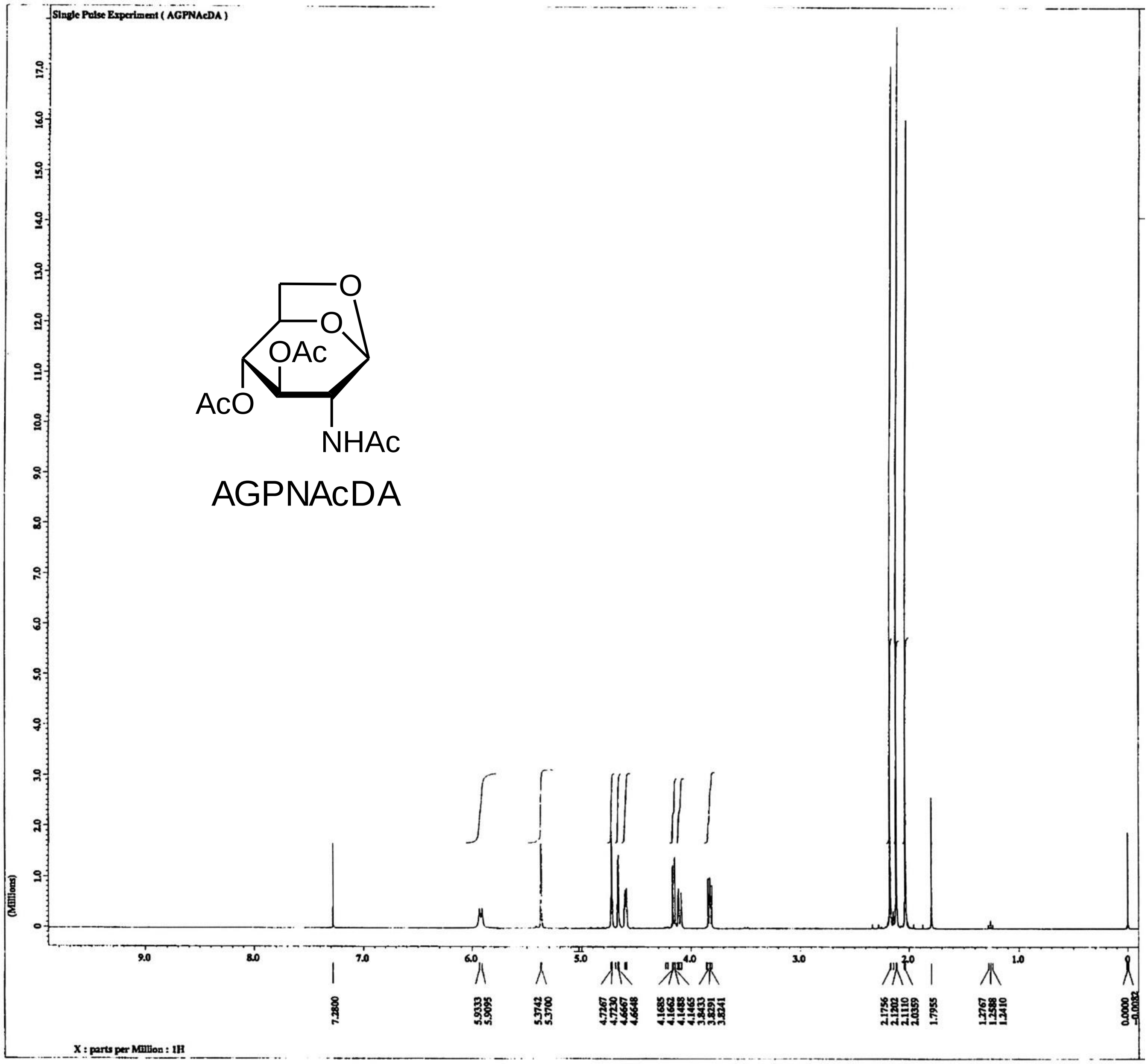

Figure S3.1: ^{1}H NMR spectrum of AGPNAcDA (400 MHz, $CDCl_3$).

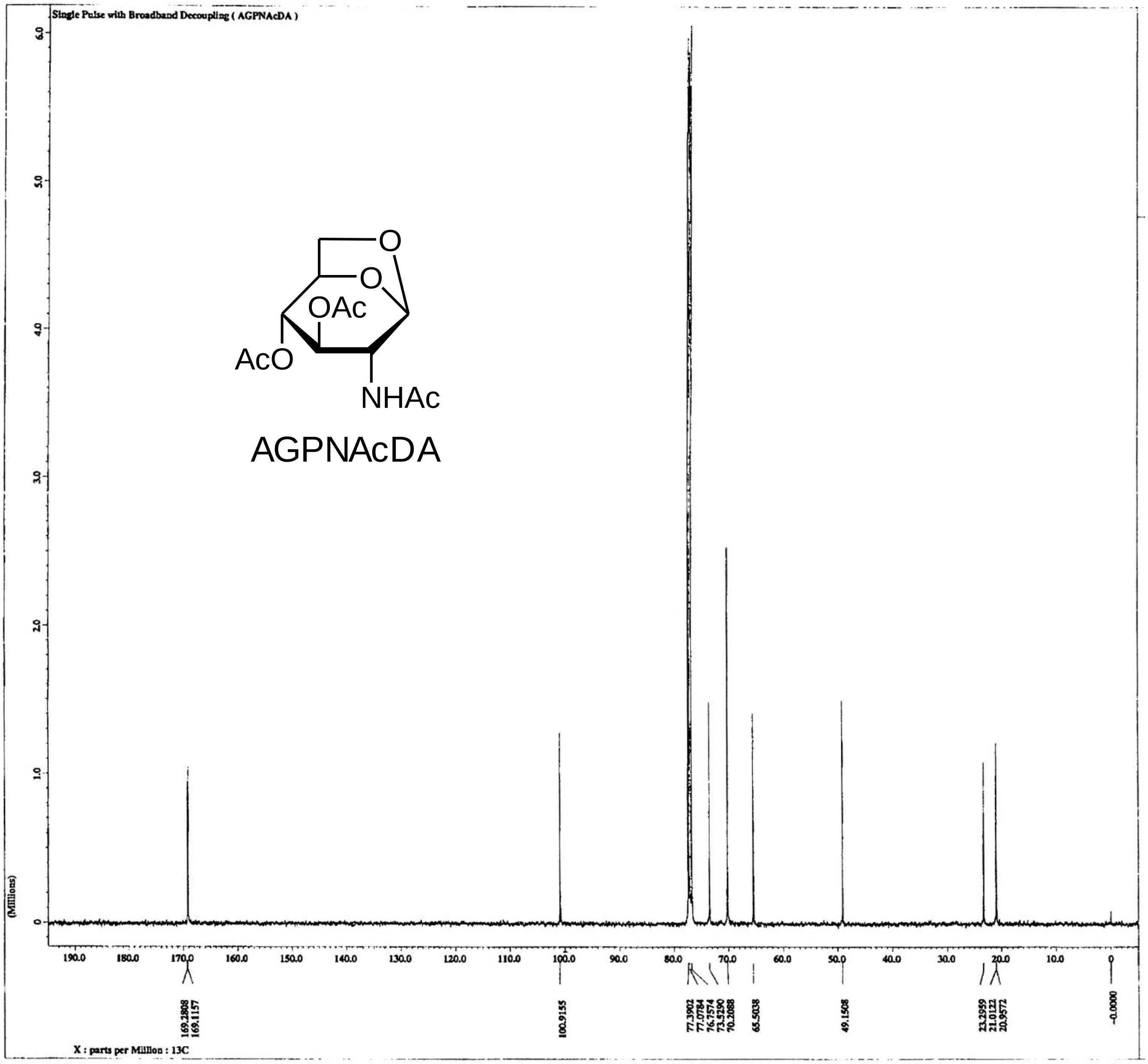

Figure S3.2: ^{13}C NMR spectrum of AGPNAcDA (100 MHz, $CDCl_3$).

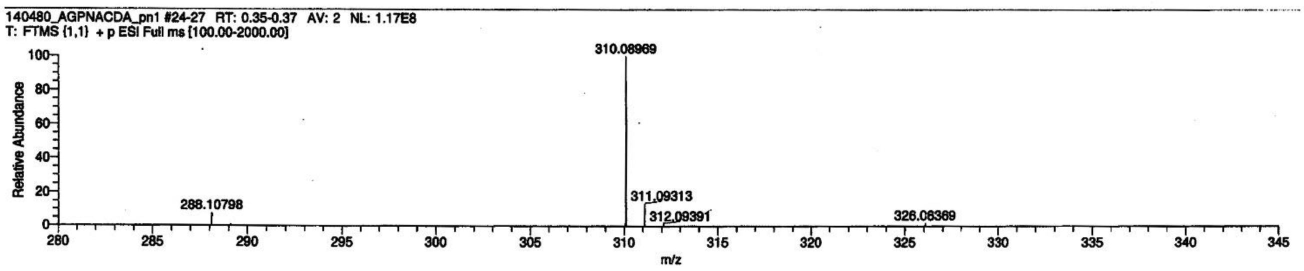

Elemental composition search on mass 310.08969

m/z= 305.08969-315.08969

Isotope	Min	Max
O-16	0	10
C-12	0	30
H-1	0	60
Na-23	0	1
N-14	0	1
B-10	0	1
S-32	0	1

Charge 1
Mass tolerance 5.00 ppm
Nitrogen rule not used
RDB equiv -1.00-100.00
max results 100

m/z	Theo. Mass	Delta (ppm)	RDB equiv.	Composition
310.08969	310.08972	-0.11	4.5	$C_{12}H_{17}O_7NNa$
	310.08963	0.21	11.5	$C_{18}H_{16}O_2NS$
	310.08945	0.78	3.0	$C_{11}H_{18}O_{10}$
	310.08863	3.43	12.0	$C_{18}H_{13}O_3{}^{10}BNa$
	310.09103	-4.33	15.0	$C_{20}H_{12}O_3{}^{10}B$

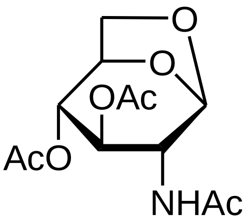

AGPNAcDA

Figure S3.3: ESI-HRMS of AGPNAcDA along with analytical data.

4. Supporting data for AGFNAcDA

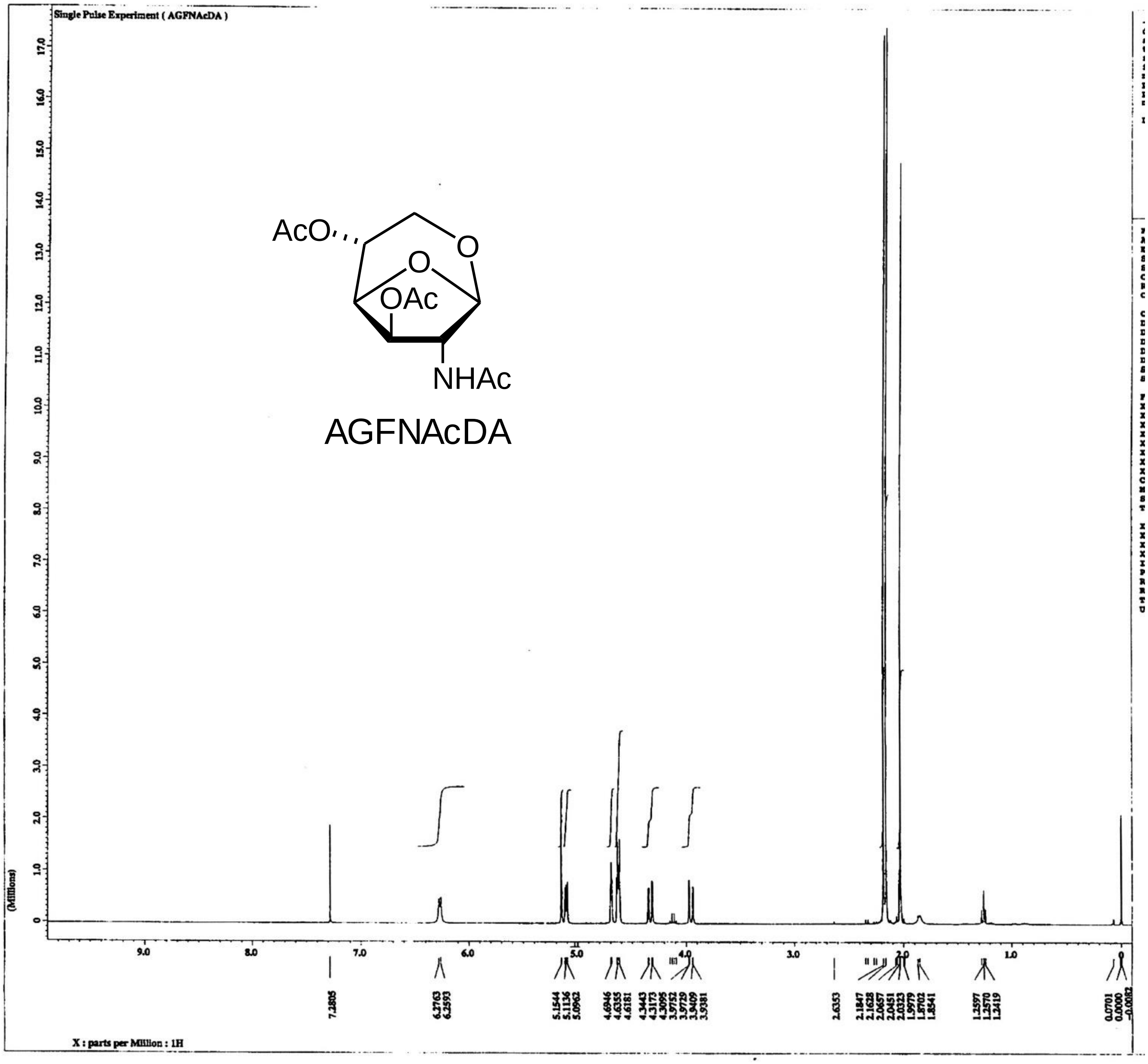

Figure S4.1: ^{1}H NMR spectrum of AGFNAcDA (400 MHz, $CDCl_3$).

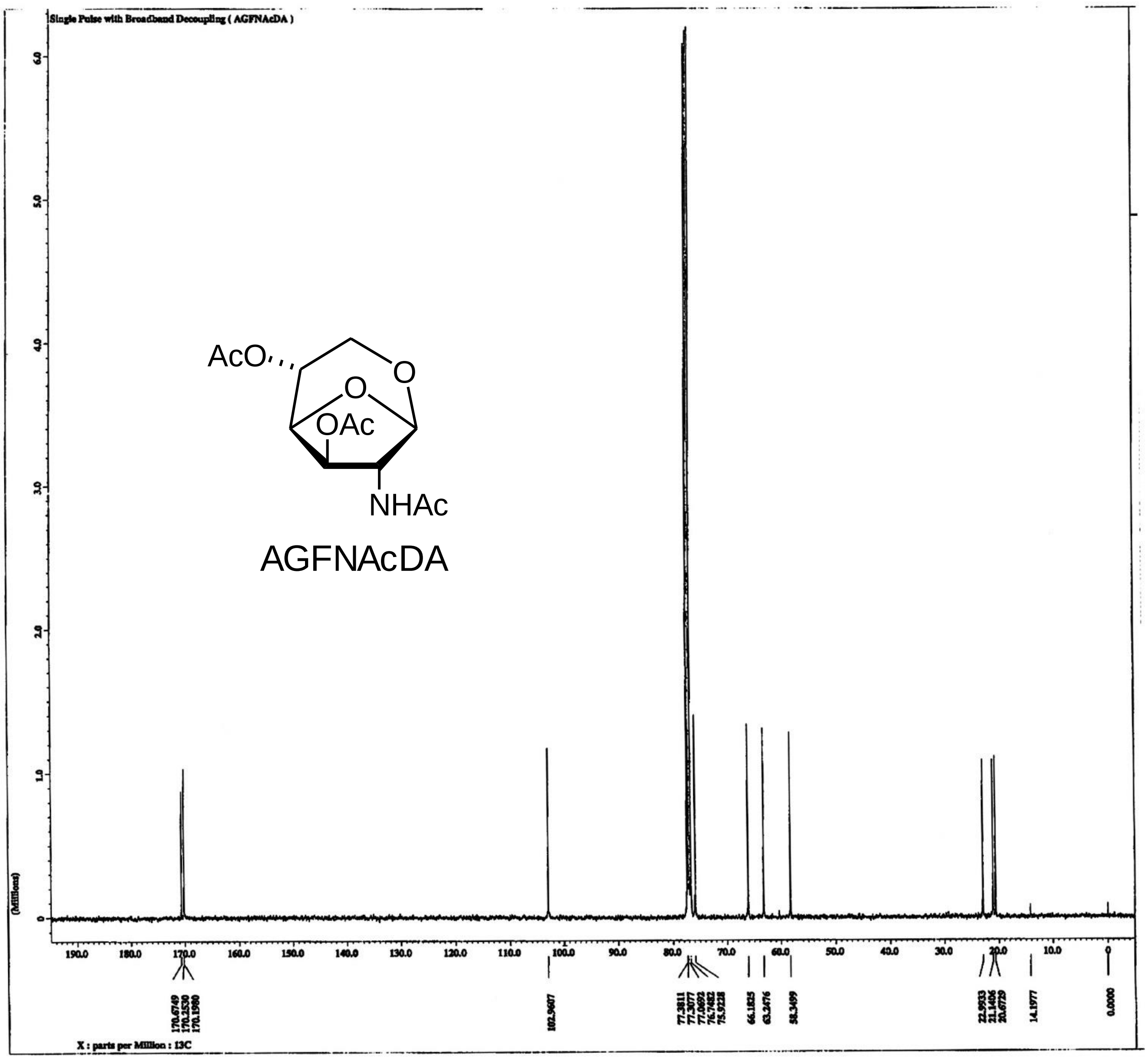

Figure S4.2: ^{13}C NMR spectrum of AGFNAcDA (100 MHz, $CDCl_3$).

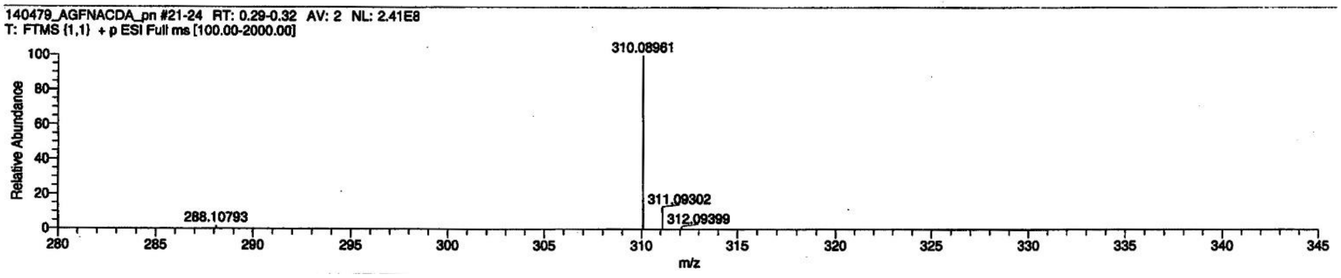

Elemental composition search on mass 310.08965

m/z= 305.08965-315.08965

Isotope	Min	Max
O-16	0	10
C-12	0	30
H-1	0	60
Na-23	0	1
N-14	0	1

Charge 1
Mass tolerance 5.00 ppm
Nitrogen rule not used
RDB equiv -1.00-100.00
max results 100

m/z	Theo. Mass	Delta (ppm)	RDB equiv.	Composition
310.08965	310.08972	-0.24	4.5	$C_{12}H_{17}O_7NNa$
	310.08945	0.65	3.0	$C_{11}H_{18}O_{10}$

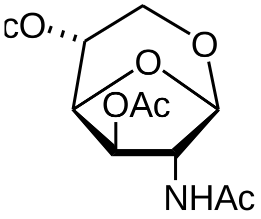

AGFNAcDA

Figure S4.3: ESI-HRMS of AGFNAcDA along with analytical data.
